# Hydrothermal liquefaction of microalgae over transition metal supported TiO_2_ catalyst

**DOI:** 10.1016/j.biortech.2017.11.051

**Published:** 2018-02

**Authors:** Wenjia Wang, Youtong Xu, Xiaoxiao Wang, Bokun Zhang, Wenying Tian, Jinglai Zhang

**Affiliations:** a School of Environment and Natural Resources, Renmin University of China, Beijing 100872, PR China; b Sinosoft Company Limited, Beijing 100190, PR China; c College of Life Science and Technology, Harbin Normal University, Harbin, Heilongjiang 150025, PR China

**Keywords:** Hydrothermal liquefaction, Microalgae, Catalyst, Biocrude, Ni/TiO_2_

## Abstract

Hydrothermal liquefaction (HTL) of microalgae *Nannochloropsis* (NAS) over various transition metal M/TiO_2_ (M = Fe, Co, Ni, Mo, and Mn) was investigated. Ni/TiO_2_ was the most effective catalyst to improve the yield and quality of biocrude and the liquefaction conversion. Ni/TiO_2_ was characterized by XRD, XRF, and XPS. The research of Effect of reaction temperature on HTL of NAS over Ni/TiO_2_ suggested that 300 °C led to a maximum biocrude yield of 48.23% and the highest liquefaction conversion of 89.28%. Adding Ni/TiO_2_ catalyst reduced the viscosity and provided more light-fraction in biocrude while brought a slight increase in total acid number (TAN). Gas chromatography-mass spectrometry (GC-MS) analysis demonstrated that adding Ni/TiO_2_ considerably changed the composition of biocrude and the possible pathways were discussed. Reproduction test showed the Ni/TiO_2_ has an excellent reproduction ability in HTL of NAS.

## Highlights

• Ni/TiO_2_ improves the hydrothermal liquefaction of *Nannochloropsis*.
• A reaction temperature of 300 °C leads to the best liquefaction effect.
• The viscosity, TAN, and boiling point distribution of biocrude changed with Ni/TiO_2_.
• Adding Ni/TiO_2_ results in more acids and hydrocarbons in biocrude.
• The Ni/TiO_2_ catalyst performs a good reproduction ability.

## 1 Introduction

Most of the primary energy supply nowadays comes from the finite fossil fuel, and this situation has caused the severe energy crisis and endless pollution (Yeh et al., 2013). Biomass, as the appropriate replacement of fossil fuel for many advantages, has drawn great attention (McKendry, 2002a). As the third generation of biomass, microalgae is a potential biomass resource, because they are with a higher energy content, can use greenhouse gas CO_2_ while growing, and grow fast (Lam and Lee, 2012). Within various technical methods for the algae conversion, hydrothermal liquefaction (HTL) is widely considered as a promising thermochemical conversion process (Elliott, 2016, McKendry, 2002b).

HTL is a thermochemical process which carried out at high temperature (200–400 °C), and high pressure (6–15 MPa) in an oxygen-free and thermal-isolated reactor. The process can directly convert the wet biomass into high-energy-dense biofuel biocrude (Tian et al., 2014). The HTL process requires a lower operating temperature than that of pyrolysis and gasification, which leads to a lower equipment requirement and less energy input. Moreover, the water in wet microalgae is both the solvent and reaction medium in HTL process, which means no extra operating cost for feedstock drying (Goyal et al., 2008). Nonetheless, the non-catalytic HTL (direct HTL) of microalgae still presented the improvement space in biocrude yield, and the biocrude is with higher heteroatom content (N, O, and S) than petroleum [10–12]. Therefore, screening the appropriate algae liquefaction catalyst for efficient liquefaction and heteroatom content reduction has drawn great attention.

Various homogeneous catalysts including acids, alkalis, and metal salts were applied in the HTL of microalgae and showed a positive effect on biocrude yield or biocrude quality (Dote et al., 1994, Jena et al., 2012, Minowa et al., 1995, Shakya et al., 2015). However, the homogeneous catalyst is hard for separation and reuse in industrial application, and the heterogeneous catalysts should be the substitution. Molecule sieves, rare metals, transition metal oxides and supported metal catalysts have been widely applied in catalytic liquefaction of microalgae (Chen et al., 2017, Chen et al., 2015b, Fu et al., 2011, Jena et al., 2012). Among these catalysts, the transition metals like Ni, Co, Fe or Mo, could reduce the heteroatom (O, N, and S) content in biocrude, or increase the biocrude yield(Biller et al., 2011, Duan and Savage, 2011b, Rojas-Perez et al., 2015, Zhang et al., 2013). The transition metals could be the active component in the microalgae liquefaction catalyst. However, more works are required for screening active ingredient and assembling appropriate catalyst composition.

On the other hand, the HTL process takes place in the high-temperature and high-pressure environment. The HTL catalyst must present a high thermostability and be easy in reproduction. That requires a proper catalyst support (Banares, 1999, Schwarz et al., 1995). Titanium Oxide (TiO_2_), as a widely accepted support for industrial production and technical research due to its high thermal stability and activity in oxidation-reduction catalysis (Aranda-Perez et al., 2017, Chen et al., 2015a, Choy et al., 1998). Therefore, TiO_2_ might be a potential catalyst support applied in HTL of microalgae. At present, there is limited literature involving the HTL of microalgae with TiO_2_-supported transition metal as the catalyst. This research would fill this gap better.

In this paper, a series of transition metal (Fe, Co, Ni, Mo, Mn) supported TiO_2_ catalysts were applied in the HTL of microalgae. We evaluated the effect of different catalysts on the liquefaction conversion of microalgae, the yield of main product biocrude as well as the element content of biocrude samples. Catalysts were characterized by X-ray photoelectron spectroscopy (XPS), X-ray fluorescence (XRF) and X-ray diffraction (XRD). The effect of operating conditions reaction temperature on the HTL process was investigated with the best catalyst Ni/TiO_2_. Detailed characterization of biocrude was taken, and different properties like viscosity, boiling point distribution, total acid number (TAN), and molecular identification were studied while the possible reaction pathways during the HTL process were discussed. Moreover, the reproduction ability of the Ni/TiO_2_ catalyst was also investigated.

## 2 Materials and methods

### 2.1 Materials

The feedstock *Nannochloropsis* (NAS) was provided by Shandong Yantai HaiRong Biology Technology Co., Ltd (Shandong, China). The feedstock was mashed, sieved at 80-mesh sieve and then dried at 105 °C for 24 h before use. The ash content of the NAS was 6.8%, and the contents of its C, H, O, N, and S elements were determined to be 47.08, 8.77, 34.54, 8.07 and 1.54%, respectively. The lipid, protein and carbohydrate contents in the organic matters of NAS were 23.2, 66.5 and 10.3%, respectively. Titanium dioxide (TiO_2_) powder and transition metal nitrates were purchased from Aladdin Reagent Co. LTD (Shanghai, China). All chemicals used were of analytical reagent grade without further purification. All the water used in experiments was deionized water.

### 2.2 Preparation and characterization of the catalyst

Excessive impregnation method was used for catalyst preparation to ensure plenty of transition metal was supported on the TiO_2_. Transition metal nitrates [Fe(NO_3_)_2_, Co(NO_3_)_2_, Mo(NO_3_)_3_, Mn(NO_3_)_2_ and Ni(NO_3_)_2_ ] were dissolved into deionized water to the setting theoretical concentration of 0.75 mol/L. Then the impregnation solution (100 mL) was dropped onto TiO_2_ powder (20 g) with continuous stirring in a 250 mL beaker at room temperature (25 °C). After the metal permeated into the support for 24 h at room temperature, the impregnation liquid was separated out by decantation, and then the impregnation product was dried at 110 °C for 12 h, followed by a calcination process in a muffle oven at 600 °C for 4 h. The calcinated catalyst was sieved at 80-mesh sieve and stored in a drying vessel before HTL experiments. The obtained catalyst was labeled as M/TiO_2_, where M = Fe, Co, Mo, Mn, and Ni.

XRF analysis was operated on an ARL ADVANT’X instrument, using Rh Kα radiation operating at 3600 W. XRD patterns were measured by a Ultima IV (Rigaku, Japan) to examine the phase composition, with a Cu Kα source (λ = 0.1541 nm, voltage = 40 kV, current = 40 mA, scanning g step length = 0.02°). The scanning angle range was from 5 to 90°. The X-ray tube was operated at 60 kV and 120 mA. Catalyst samples were analyzed by XPS measurements conducted with a PHI Quantera microprobe (ULVAC-PHI Inc., Japan), which equipped with an Al anode as the monochromatized X-ray source (1486.7 eV run at 10 kV and 15 mA in fixed analyzer transmission mode) to investigate the surface composition and the valence state. The binding energies were calibrated concerning the signal for adventitious carbon (binding energy, 284.6 eV). The peak fitting procedure was performed with XPS Peak 4.1.

### 2.3 Catalytic liquefaction procedure

The HTL process was carried in a batch high-pressure stainless steel reactor with 1800 ml capacity (Parr Instruments Co., Moline, PA, USA). In a typical liquefaction experiment, 120 g of NAS was added into the reactor and mixed with 480 g of water. The dosage of catalyst was equal to 10 wt% of microalgae (12 g). After loading the feedstock, the reactor was sealed, and N_2_ was pumped into the reactor for three times to expel the air in the reactor, and then the sealed reactor was heated to the desired temperature and held for 30 min. The reaction pressure is approximately 6 ∼ 8 MPa during the liquefaction process.

After the holding time finished, the reactor was cooled down to room temperature (25 °C) and decompressed through the gas outlet tube. Separation of catalytic liquefaction products was carried out as follows. Gaseous products were vented directly during decompressed. The other phase products were poured out, and the reactor was washed for at least three times with 400 mL of dichloromethane (DCM). The products and DCM washing solution were collected. The solid residue (SR) was separated from the liquid mixture through filtration. The SR was dried at 105 °C for 24 h to constant weight and then weighed to calculation the liquefaction conversion. The SR was consisted with ash in the algae, the catalyst, and the solid organic matters. The liquid mixture was divided into water-soluble phase and DCM-soluble phase in a separating funnel. The DCM was removed from the DCM-soluble phase in a rotary evaporator at 60 °C under reduced pressure (0.05 MPa). The obtained DCM-removed black sticky liquid was defined as the aim product biocrude and weighted to calculate the biocrude yield. The analysis of the gaseous and water-soluble phase product is beyond the scope of this paper. In this research, the experiments carried out in the absence of catalyst was designated as blank and used for comparison. The presented data from liquefaction experiments were the average values based on parallel experiments repeated for at least three times and the errors presented were the standard deviations.

### 2.4 Analytic methods of the biocrude

An Elemental Analyzer (Vario EL cube Elementar, Hanau, Germany) analyzed the elemental composition (C, H, N, and S) of biocrude. All other elements were approximated to be zero, and the content of oxygen (O) was determined by difference. The higher heating values (HHV) of the feedstock and the biocrude were calculated using the formula based on the elemental compositions. Each sample was tested for three times and presented the average value.

The viscosity of the biocrude was measured based on a modified ASTM-D 2983-2017. Biocrude samples were analyzed by a viscometer (Brookfield, DV-III, USA). The use of higher temperatures than the standard was due to the very high viscosity of biocrude at low temperature. A circulating bath with ethylene glycol as the coolant was used to keep constant temperatures from 10 to 60 °C with an accuracy of 1 °C. The viscosity measurements were replicated three times and showed the average value. Samples were analyzed for viscosity at 40 and 60 °C, respectively. The standard errors of experimental results were less than ± 1%.

The thermal gravimetric analysis (TGA) was carried out on a TG analyzer (DTG-60, Shimadzu, Japan). Each sample (10 ± 0.5 mg) was heated from 50 to 500 °C at a heating rate of 10 °C min^−1^ in pure nitrogen with a flow rate of 50 ml min^−1^. Each experiment was replicated for at least three times to ensure reproducibility and presented the average values, and the standard errors of experimental results were within ± 2%.

The total acid number (TAN) was measured by a modified European standard EN 14104 to be able to use color indicator phenolphthalein even the biocrude is black, as reported by Anouti et al. (2016). To measure the TAN of biocrude sample, 25 mg of biocrude was dissolved in 5 mL of isopropyl alcohol and then titrated by a potassium hydroxide (KOH) solution of 20 mM with the color indicator phenolphthalein at room temperature (25 °C). The titration process was repeated for at least three times and presented the average value.

The organic composition of biocrude was analyzed with a gas chromatography-mass spectrometry (GC-MS, QP2010, Shimadzu Co., Tokyo, Japan). GC-MS was equipped with a Varian DB-5 column (30 m × 0.25 mm × 0.25 μm). Helium was used as the carrier gas. The injection temperature and interface temperature were set at 250 °C and 320 °C. The ion source was adjusted to 200 °C. The mass spectrometer was operated in positive electron impact mode (EI) at 70 eV. Scan range of mass spectrum was in *m*/*z* of 20–650. All chromatogram peaks in spectra were compared with the electron impact mass spectrum from NIST Database (NIST11). The column temperature was set at 50 °C for 2 min, then ramped up at a rate of 10 °C/min to 120 °C and maintained for 1 min, afterward increased to 250 °C with the same heating rate and maintained for 20 min.

### 2.5 Calculation methods

The biocrude yield (%), liquefaction conversion (%), element enrichment (%), higher heat value (HHV, MJ kg^−1^) and energy recovery were calculated by the formula (1), (2), (3), (4):

(1) $$\text{biocrude yield}\;(\%)=\frac{{\text{m}}_{\text{B}}}{{\text{m}}_{\text{M}}}\times 100\%$$

(2) $$\text{liquefaction conversion}\;(\%)=\left(1-\frac{{\text{m}}_{\text{SR}}-{\text{m}}_{\text{A}}-{\text{m}}_{\text{C}}}{{\text{m}}_{\text{M}}}\right)\times 100\%$$

(3) $$\text{element enrichment}\;(\%)=\frac{\text{biocrude yield}\times {\text{E}}_{\text{B}}}{{\text{E}}_{\text{M}}}$$

(4) $${\text{HHV(MJ kg}}^{-1})=0.3404{\text{C}}_{\text{B}}+1.2432{\text{H}}_{\text{B}}+0.0628{\text{N}}_{\text{B}}+0.1909{\text{S}}_{\text{B}}-0.0984{\text{O}}_{\text{B}}$$

where m_B_, m_M_, m_SR_, m_A_, m_C_ is the mass of biocrude, microalgae, solid residues, ashes in microalgae and the catalyst, respectively. E_B_ and E_M_ are the element content (%) in biocrude and microalgae (E = C, H, N, O, and S).

The higher heating values (HHV) of the feedstock and the biocrude were calculated using the formula (4) based on the elemental compositions (Channiwala and Parikh, 2002).

The energy recovery (ER) was set to determine the energy efficiency of the HTL process, and ER was defined as the ratio of HHV of biocrude to HHV of NAS. The calculation of ER was based on formula (5), where the HHVB and HHVM are the higher heat values of biocrude and microalgae, respectively.

(5) $$\text{Energy recovery}\;(\%)=\frac{{\text{HHV}}_{\text{B}}\times \text{biocrude yield}}{{\text{HHV}}_{\text{M}}}$$

## 3 Result and discussion

### 3.1 Effect of catalysts on biocrude yield and liquefaction conversion

Adding appropriate catalyst in HTL process could improve the liquefaction conversion, biocrude yield, and the biocrude quality (Biller et al., 2011). Table 1 presented the effect of different transition metal/TiO_2_ catalysts on the biocrude yields and liquefaction conversions from the HTL of NAS. Other operating conditions were a reaction temperature of 270 °C, a holding time of 30 min, and a catalyst dosage of 10 wt% of microalgae.

**Table 1:** Effect of HTL of NAS over various transition metal/TiO_2_ catalyst.

	Fe	Co	Ni	Mo	Mn	Blank
Liquefaction conversion (%)	78.72	75.97	85.19	84.33	82.56	79.31
Biocrude yield (%)	29.10	32.73	42.40	35.86	31.63	30.10

*Element content (wt%)*						
Carbon	70.64	69.89	69.29	69.55	69.22	70.50
Hydrogen	9.77	9.34	9.91	8.78	8.55	9.73
Oxygen^a^	12.15	12.68	13.68	13.87	14.61	11.01
Nitrogen	7.00	7.62	6.75	7.36	7.22	7.68
Sulfur	0.44	0.47	0.37	0.44	0.40	1.08
HHV (MJ/kg)	35.47	34.67	35.00	33.72	33.24	35.65
Energy Recovery (%)	42.47	46.70	61.08	49.77	43.26	44.16
H/C atom ratio	1.66	1.60	1.72	1.51	1.48	1.66
O/C atom ratio	0.13	0.14	0.15	0.15	0.16	0.12

*Element enrichment (%)*						
Carbon	43.66	48.59	62.40	52.98	46.50	45.07
Hydrogen	32.42	34.86	47.91	35.90	30.84	33.39
Nitrogen	25.24	25.02	28.71	26.47	22.91	23.19
Oxygen	10.24	12.02	16.79	14.40	13.38	9.59

a Determined by difference.

As shown in Table 1, both the liquefaction conversion and the biocrude yield were changed by adding catalysts. The Ni, Co and Mo catalysts were conducive to improve the liquefaction conversion and biocrude yield. The results were consistent with previous literature (Duan and Savage, 2011b, Wang et al., 2017). However, the Fe and Mn catalysts showed an adverse effect while they affected the liquefaction of bagasse, which contains different biochemical content (Long et al., 2016). With Ni catalyst applied in the HTL process, the liquefaction conversion and biocrude yield peaked at 85.19% and 42.40%, respectively, which was 10% higher than the blank experiment, and performed better than any other catalysts. Moreover, the elemental analysis in Table 1 provided a significant promotion of enrichment for both carbon and hydrogen element (from 45.07 and 33.39% to 62.40 and 47.91%, respectively) by adding nickel catalyst. This trend is pleasant for promoting the biocrude more alike to the hydrocarbon fuel, as the H/C atom ratio increased from 1.66 to 1.72. According to Bill’s research, the biochemical compounds in microalgae were usually liquefied as the following orders: lipid, protein, and carbohydrate (Biller and Ross, 2011). The obtained biocrude yield was higher than the lipid content in NAS, especially after adding Ni/TiO_2_ catalyst. The improvement of biocrude yield may be attributed to the liquefaction of protein and carbohydrate, and adding nickel catalyst should have enhanced this conversion during the liquefaction process. Since the conversion of protein and carbohydrate to biocrude by direct liquefaction required higher active energy, the introduction of Ni/TiO_2_ could reduce the activation energy for the liquefaction of protein and carbohydrate, and convert more these biochemical compositions into biocrude. Based on the high nitrogen and oxygen content of protein and the high oxygen content of protein and carbohydrate, the increased enrichment of N and O (from 23.19 and 9.59% to 28.71 and 16.79%, respectively) in the biocrude after adding Ni/TiO_2_ can support this conjecture. Also, the energy recovery sharply increased from 44.16 to 61.08% with Ni/TiO_2_ catalyst, and this suggested an improved energy efficiency of HTL of NAS. Therefore, the Ni/TiO_2_ catalyst could be an efficient and appropriate catalyst for the catalytic liquefaction of NAS indeed.

### 3.2 Characterization of the Ni/TiO_2_ catalyst

As widely known, the color of the metallic oxide NiO is yellow, which was just consistent with the color of Ni/TiO_2_ catalyst and illustrated the element Ni should be supported on the TiO_2_ support. To further verify this assumption, XRF analysis was investigated and indicated that the relative contents of main elements in the Ni/TiO_2_ catalyst. The obtained results demonstrated that the Ni/TiO_2_ catalyst mainly contains Ti (58.44 wt%), O (39.61 wt%), and Ni (1.95 wt%). The XRF results suggested that the main component of the catalyst, however, could be the TiO_2_ support. The content of Ni is much less than that of TiO_2_.

The XRD analysis is widely used for characterization of crystal structure properties of the catalysts, and the XRD characterization of Ni/TiO_2_ catalyst identified that the major component in the catalyst was anatase phase TiO_2_ (PDF#21-1272).The XRD characterization of Ni/TiO_2_ catalyst identified that the major component in the catalyst was anatase phase TiO_2_ (PDF#21-1272) with a few rutile phase TiO_2_. No Ni element was found out in the XRD analysis suggested that the Ni element could exist in the form of amorphous state in the catalyst (Yao et al., 2010) and with a small amount.

Further XPS analysis of the catalyst obtained an increased Ni content from 1.95 wt% of XRF to 3.97 wt% of XPS and confirmed an enrichment of Ni element on the surface of the Ni/TiO_2_ (Liu et al., 2013). The metal element in the catalyst existed in the following state: Ti^4+^, Ni^2+^, and Ni^3+^ (Kang and Rhee, 2001, Yin et al., 2016) and suggested that Ni existed in the form of NiO and Ni_2_O_3_. The O element has at least two kinds of chemical states of chemical-adsorbed oxygen (O_H_) and crystal-lattice oxygen (O_L_) (Yao et al., 2010). The O_H_ was firmly related to the hydroxyl groups resulting mainly from the chemisorbed water, which was a favor to the catalytic reactions reported in many references (Jing et al., 2006). The peak of O_L_ could be mainly attributed to the Ti—O in TiO_2_ crystal lattice. Detailed characterization patterns were provided in the Supplement information.

### 3.3 Effect of reaction temperature on biocrude yield and liquefaction conversion

**Fig. 1:**
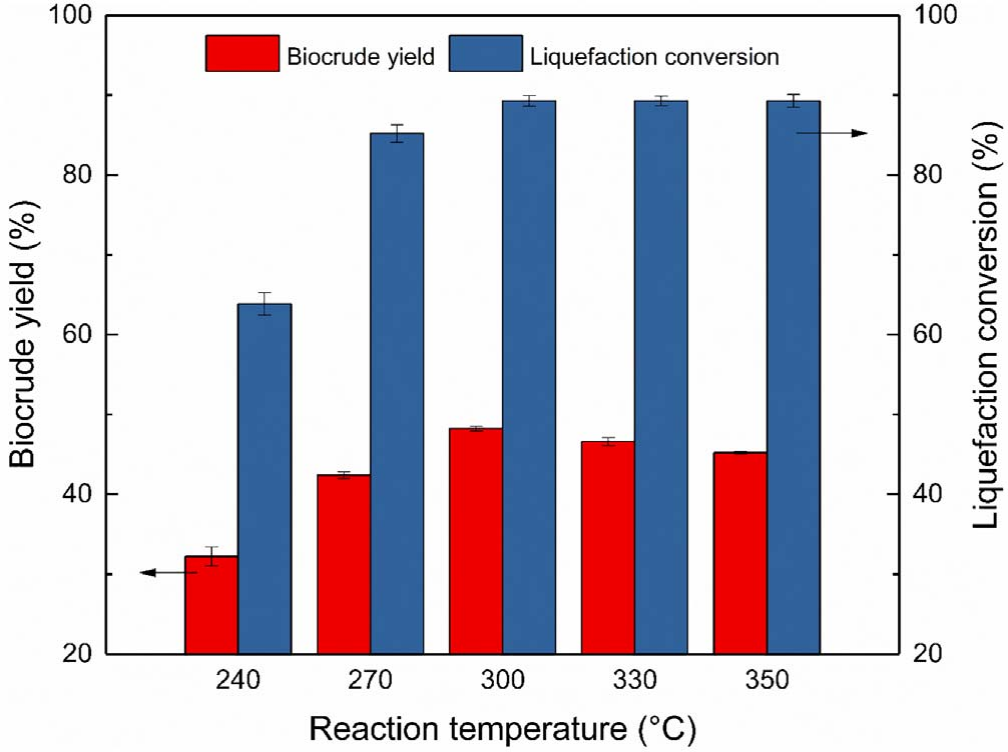
Effect of reaction temperature on HTL of NAS over Ni/TiO_2._

The high temperature of hydrothermal liquefaction provided the necessary energy to decompose the macromolecules in feedstock and produced biocrude and other byproducts. The reaction temperature showed a significant influence on the distribution of product yield and the element composition of biocrude (Muppaneni et al., 2017). An appropriate reaction temperature could improve the biocrude yield and liquefaction conversion (Anastasakis and Ross, 2011). Fig. 1 showed the effect of reaction temperature on the biocrude yield and liquefaction conversion. The liquefaction temperatures were in the range of 240–360 °C. The other operating parameters were all the same as the experiments in Section 3.1. Each liquefaction experiment was repeated for at least three times, only the average values and the standard deviation values were presented in the Fig. 1.

As shown in Fig. 1, the reaction temperature did show a remarkable influence on the HTL process. With the reaction temperature rising from 240 °C, the biocrude yield continuously increased to the highest peak of 48.23% at 300 °C. That value was higher than those from HTL of NAS without catalyst (around 40%) or with Na_2_CO_3_ catalyst (around 32%) reported by Shakya (Shakya et al., 2015). Since the result was obtained with the same reaction temperature and algae strain, additional liquefaction experiments at 300 °C without catalyst and Na_2_CO_3_ were added. The obtained biocrude yields (39.48% and 37.42%) were lower than the value with Ni/TiO_2_. The difference with Shakya’s data should come from the different operating condition like biomass loading (16–20%), holding time (1 h to 30 min), initial pressure (69–0 kPa), and the biochemical composition of the NAS used in the two studies. In the temperature range of 240–300 °C, the growth of biocrude yield could come from the conversion of non-lipid biochemical molecules like high-nitrogen-and-oxygen-content proteins and high-oxygen-content carbohydrates, which need more energy to crack and convert into biocrude(Valdez et al., 2014), as the liquefaction conversion increased from 63.85 to 89.28%. The increase is also corresponding to the element analysis that the oxygen and nitrogen content in the biocrude increased with a higher temperature in this temperature range of 240–300 °C. With further increase of reaction temperature to 360 °C, the biocrude yield presented a slight but gradual decrease to 45.21% while the liquefaction conversion remained steady. The decrease of biocrude yield could be due to the partly cracking of biocrude into small molecules no longer captured in the oily fraction (Valdez and Savage, 2013). Therefore, a reaction temperature of 300 °C could be appropriate for the HTL of NAS over the Ni/TiO_2_ catalyst.

### 3.4 Analysis of the biocrude with/without the Ni/TiO_2_ catalyst

#### 3.4.1 Viscosity of biocrude

As the representation of the fluidity of liquid fuel, the viscosity is an important measurement parameter to evaluate the quality of the biocrude. A lower viscosity leads to a better fluidity for the further refining and transportation of biocrude fuel. In this section, the viscosities of the biocrude with/without Ni/TiO_2_ catalyst at 300 °C were measured and discussed.

At lower temperature, the biocrude could flow freely but was highly viscous. However, biocrude samples all showed a better fluidity under higher temperature. According to the viscosity test, the viscosity of biocrude samples was improved from 214.3 (40 °C) and 68.73 cP (60 °C) to 187.1 (40 °C) and 48.31 cP (60 °C) by adding Ni/TiO2 catalyst. As the viscosity is largely influenced by the amount of heavy components (including polymers) in biocrude, the less-viscosity catalyzed biocrude should contain less heavy-oil content. This improvement is welcome for future application of biocrude in fuel refining and direct utilization. The obtained viscosities of biocrude are closed to that from HTL of Spirulina, but still much higher than that from pyrolysis bio-oil and petroleum crude (Jena and Das, 2011). Therefore, the biocrude from HTL of NAS over Ni/TiO_2_ catalyst would require more robust fuel injectors for engine use and should be upgraded to be more alike commercial fuel.

#### 3.4.2 TGA of biocrude

The thermal gravimetric analysis (TGA) has been widely used as a miniature distillation process in analyzing the biocrude and bio-oil from thermochemical conversion. Although there could be thermal degradation in the heating process, the TGA can still provide an estimate of the boiling point distribution of the biocrude (Anastasakis and Ross, 2011). The boiling point distribution of biocrude samples with/without Ni/TiO_2_ catalyst at 300 °C was listed in Table 2.

**Table 2:** the boiling point distribution of biocrude samples obtained with/without Ni/TiO_2_ catalyst (300 °C, 30 min).

Boiling point range (°C)	Biocrude fraction	
	With Ni/TiO_2_	Blank
50–150	25.29	26.73
150–200	18.44	14.86
200–250	13.38	14.20
250–300	11.86	10.76
300–350	6.66	3.15
350–500	12.06	15.71
>500	12.31	14.59
Total fraction (%) of light fraction (boiling point <350 °C)		
	75.63	69.70

As shown in Table 2, the distillation range of 150–500 °C was the major weight loss interval for both samples. It is evident that the dosage of Ni/TiO_2_ catalyst did influence the boiling point range of the biocrude, as the biocrude obtained with catalyst had more distribution at 50–300 °C but less distribution at 350–500 °C. A pleasant change was that the light fraction with a boiling point less than 350 °C in biocrude increased from 69.70 to 75.63% of the total fraction, by adding the catalyst. The result indicated that the Ni/TiO_2_ catalyst is favorable to produce finer biocrude for further separation and refining to Commercial fuels and chemicals (Huang et al., 2014). The increasing light fraction might be associated with the improvement of viscosity after the dosage of Ni/TiO_2_ catalyst. However, the results in Table 2 also demonstrated that the biocrude still contained a certain amount of high-boiling-point compounds and these cannot be analyzed by GC-MS. Therefore, further refining of the biocrude should be taken in the future.

#### 3.4.3 TAN of the biocrude samples

Total acid number (TAN) is a measurement of acidity that is determined by the amount of potassium hydroxide in milligrams that are needed to neutralize the acidic components in one gram of oil. It is an important measurement parameter to evaluate the quality of crude petroleum oil. A high value of TAN could cause damage to machinery and storage facility. This parameter has been widely used to determine and analyze the property of biodiesel and pyrolysis oil and could apply to measure the acidity of biocrude from HTL. In this section, the TAN of biocrude obtained at 300 °C with/without Ni/TiO_2_ catalyst was measured.

According to the result, the TAN values are much higher than that from the standard of commercial fuel (usually specified no more than 0.5 mg KOH g^−1^) (Duan and Savage, 2011a). Comparing the TAN of biocrude samples, the TAN value increased from 234.65 to 268.68 mg KOH g^−1^ by adding Ni/TiO_2_ catalyst. The TAN of biocrude form HTL of NAS without the Ni/TiO_2_ catalyst is much higher than that from Shakya et al. (2015) with acetone extraction solvent but is similar to that reported by Duan and Savage (2011a). As the TAN could directly reflect the amount of carboxyl groups in the sample, the increase of TAN could be due to the more compounds with carboxyl functional groups, like fatty acid, carboxylates, and amino acids. According to the biochemical content of the feedstock NAS, the fatty acid could come from the hydrolysis of lipid while the hydrolysis of protein produced the amino acid. Further internal reactions could product these carboxylates. Another origin of the higher TAN could be the phenolic matters, which could come from the conversion of amino acids. It seems that although the Ni/TiO_2_ catalyst increased the biocrude yield and liquefaction conversion of NAS, the unpleasant increase of TAN was obtained. Therefore, more works to reduce the TAN of the biocrude need to be done in the future.

#### 3.4.4 The GC-MS characterization of biocrude samples

**Fig. 2:**
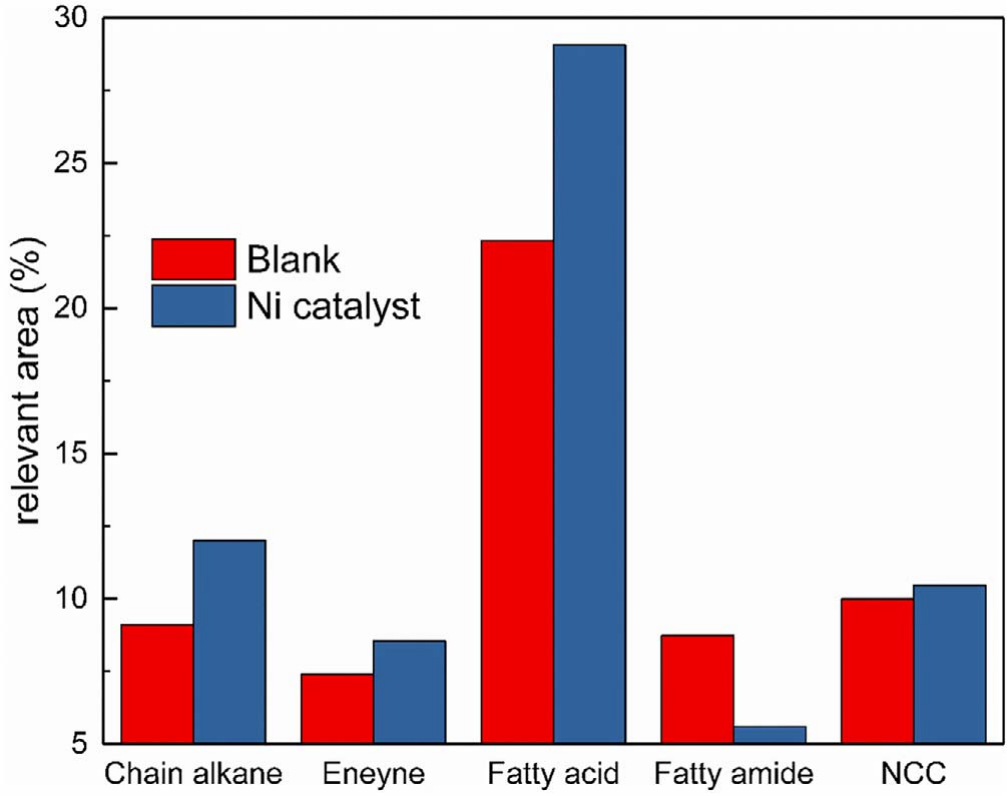
Group distribution of identified compounds from biocrude obtained with/without Ni/TiO_2_ catalyst NCC: N-containing heterocyclic compounds. NCC: N-containing heterocyclic compounds.

The biocrude obtained from the hydrothermal liquefaction of microalgae is a complex mixture of various compounds. GC-MS was widely used to identify the molecular information of the components in the biocrude. It should be noted that the identified percent only represented the compounds that can vapor in the GC gasify room and pass through the GC column. Those high-boiling-point components and thermolabile molecules might not be identified by GC-MS. According to the TG analysis in Section 3.4.2 and the GC-MS operating conditions, about 30–40% of the compounds in biocrude could not vapor and be identified. Nevertheless, the GC-MS identification results could provide an approximate molecular characterization of the aimed liquefaction product biocrude. To simplify the discussion, the main compounds in biocrude identified by GC-MS could be classified into five groups: chain alkane, eneyne, fatty acid, fatty amide and N-containing hetero-atom compound. Individual chemical components were classified, and the sum of relative percentages of chemical components was taken into calculation. Fig. 2 presented the distribution of the chemical compounds identified by GC-MS in the biocrude samples with/without Ni/TiO_2_ obtained with a reaction temperature of 300 °C.

As shown in Fig. 2, adding Ni/TiO_2_ catalyst increased the content of chain alkanes, fatty acids and N-containing heterocyclic compounds by 9.88%, 30.20%, and 4.70%, respectively. The significantly increased amount of fatty acid could be responsible for the little higher oxygen content of biocrude. Moreover, it could respond to the increased TAN of biocrude. On the contrary, the content of fatty amides in biocrude was reduced by 35.74% with the catalyst, which could explain the decrease of nitrogen content in biocrude. Apparently, the nickel catalyst not only improved the biocrude yield and liquefaction conversion but also substantially changed the composition of biocrude. Further discussion about the possible reaction pathways would be presented in Section 3.4.5.

#### 3.4.5 Possible reaction pathways during the catalytic liquefaction process

The possible reaction pathways in direct hydrothermal liquefaction of microalgae have been detailed discussed in the previous literature (Peterson et al., 2010, Valdez and Savage, 2013). It was widely believed that there are at least three steps during the HTL process. The first step was the hydrolysis of biomass main biochemical composition of lipids, proteins, and hydrocarbons to small active molecule compound like fatty acids, amino acids, and monosaccharides. Next, reactions took place between these hydrolysis products to reconstitute liquefaction product like biocrude. Finally, cracking and re-polymerization reaction last until the HTL process stopped. According to the identification of biocrude, the nitrogen-containing compounds, which were apparently from liquefaction products of protein, can divide into two main existence forms: fatty amide and N-containing heterocyclic compounds. The fatty amide could come from the amination reaction between fatty acid and amine from the hydrolyzed amino acid (Duan et al., 2010, Liang et al., 2016, Luo et al., 2015). On the contrary, the N-containing heterocyclic compounds might be synthesized by cyclization reaction between amino acids. It should be noted that a special reaction product appeared in the biocrude, the 3-benzyl-6-isobutylpiperazine-2, 5-dione, happened to be the intermolecular dehydration-cyclization production between valine and phenylalanine. The other pathway to product N-containing heterocyclic compounds could be through the Maillard reaction (Gai et al., 2015, Zhang et al., 2016). There should be a competitive relationship between the syntheses of the two kinds of N-containing compounds, which both consumed the amino acids from the hydrolysis of proteins in NAS. With adding Ni/TiO_2_ catalyst, the scale shifted to the N-containing heterocyclic compounds. This deduction could explain the increase of fatty acids and the decrease of the fatty amide with the N-containing heterocyclic cycle compounds in the biocrude. It seems that the catalyst could impede the formation of amides. Meanwhile, the synthesis of N-containing heterocyclic compounds enhanced the conversion of less active protein and hydrocarbon into biocrude, which might be the reason why the liquefaction conversion and biocrude yield increased. Moreover, the unconsumed fatty acid could be transformed into chain alkanes or eneynes by decarboxylation (Al Alwan et al., 2015, Miao et al., 2016), like heptadecane, hexadecane, and tetradec-3-ene, which is responding to the group fraction changes in Fig. 2.

### 3.5 Reproduction test of the Ni/TiO_2_ catalyst

In this section, the reproduction ability of the Ni/TiO_2_ catalyst was examined. The biocrude yield was used to evaluate the reproduction ability of the catalyst. The fresh Ni/TiO_2_ catalyst was pre-treated in hot pressure water or reused for several times in the HTL of NAS, respectively. Two sets of the catalyst control group were applied in the reproduction test. In group A, the Ni/TiO_2_ catalyst was treated in hot water for 720 min at 270, 330, and 390 °C, respectively. In group B, the fresh catalyst was reused for catalytic liquefaction of microalgae with nine runs. Before the reuse process, the catalyst was calcinated at 600 °C in a muffle oven to remove the residue organic matters. The other liquefaction conditions were a reaction temperature of 270 °C, a holding time of 30 min, and a catalyst dosage of 10 wt% of microalgae. Table 3 presented the effect of different catalyst conditions on the yield and element composition of biocrude.

**Table 3:** Performance of the Ni/TiO_2_ catalyst in the reproduction experiments.

Catalyst condition	Biocrude yield (%)	Element content (%)				
		C	H	O^a^	N	S
Fresh catalyst	42.40	69.29	9.91	13.68	6.75	0.37

Hydrothermal treatment						
Treating temperature (°C)						
270	42.45	69.35	9.87	13.54	6.82	0.42
330	42.38	69.28	9.89	13.9	6.68	0.25
390	42.42	69.42	9.72	13.99	6.54	0.33

Reuse test						
Reused run (times)						
1	42.43	69.31	9.37	14.24	6.54	0.54
2	42.39	68.83	9.68	14.25	6.89	0.35
5	42.41	69.35	9.75	13.4	7.01	0.49
10	40.57	69.29	9.91	13.68	6.75	0.37

*Note*: Triplicate was conducted for element analysis, the relative standard deviation value was less than 1%, and only average value was presented.

a Determined by difference.

As shown in Table 3, the biocrude yield of the catalytic liquefaction of NAS over Ni/TiO_2_ catalyst pre-treated at different hydrothermal conditions hardly changed. Apparently, the hot pressure water treatment showed no significant influence on the biocrude production. According to further element analysis of the obtained biocrude, the contents of the main organic elements (C, H, O, N, and S) were unchanged almost. The Ni/TiO_2_ catalyst presented a steady catalytic effect on the HTL of NAS after the hydrothermal pre-treatment. It was demonstrated that The Ni/TiO_2_ catalyst performed a promising reproduction ability in the high-temperature environment.

The catalytic liquefaction tests with the catalysts in the group B further examined the reproduction ability of the reused Ni/TiO_2_ catalyst. According to Table 3, the biocrude yield remained unchanged almost for the first six runs and then had a slight decrease from 42.40 to 40.57% after the last five runs, compared with the fresh catalyst. The changing trend of biocrude yield indicated that the reproduction ability of the Ni/TiO_2_ catalyst performed well and only diminished a little with many times of reuse. The slight activity loss of Ni/TiO_2_ catalyst could come from the coating effect of solid residue’s inorganic component, the organic matters or deposited carbon, as the catalyst though ten runs showed a darker color the fresh catalyst, which is light yellow. However, the solid organic matters and carbon deposition have been removed in the calcination process before reuse, the change of catalyst color should be the inorganic ashes from the NAS. The XRF analysis of the catalyst reused for ten times showed that the Silicon (Si), Sulfur (S), and Magnesium (Mg) appeared in the catalyst. The coating could reduce the surface area and active site of the Ni/TiO_2_ catalyst, resulting in the decrease of biocrude yield. A similar result was obtained in the catalytic hydrothermal liquefaction of rice straw over CuZnAl catalyst (Zhou et al., 2016). Further analysis of the catalyst and discussion about the catalysis with the coating is beyond our research scope but should be given in the future.

## 4 Conclusion

Hydrothermal liquefaction of Nannochloropsis over various transition metal/TiO_2_ catalyst was investigated, and Ni/TiO_2_ promoted the biocrude yield, liquefaction conversion most. A reaction temperature of 300 °C led to the highest biocrude yield of 48.23% and the maximum liquefaction conversion of 89.28%. Adding Ni/TiO_2_ significantly reduced the viscosity, brought more light-fractions and a slight increase of TAN, and changed the molecular composition of biocrude. GC-MS suggested there were more fatty acids, alkanes, enynes by adding Ni/TiO_2_. The Ni/TiO_2_ catalyst also presented a good reproduction ability after hydrothermal treatment and reuse.
