# Supplementary material for: Hydrothermal liquefaction of microalgae over transition metal supported TiO2 catalyst
Source: Bioresour Technol. 2018 Feb;250:474–80. doi: 10.1016/j.biortech.2017.11.051 (PMC5858874; doi:10.1016/j.biortech.2017.11.051)
Supplement: Supplementary data [file mmc1.docx]

# Supplyment Information



Fig

.S1 (a) XRD pattern of the Ni/TiO2 catalyst

Fig.S1 (b) XPS spectrum of Ti_2p_ from the Ni/TiO_2_ catalyst


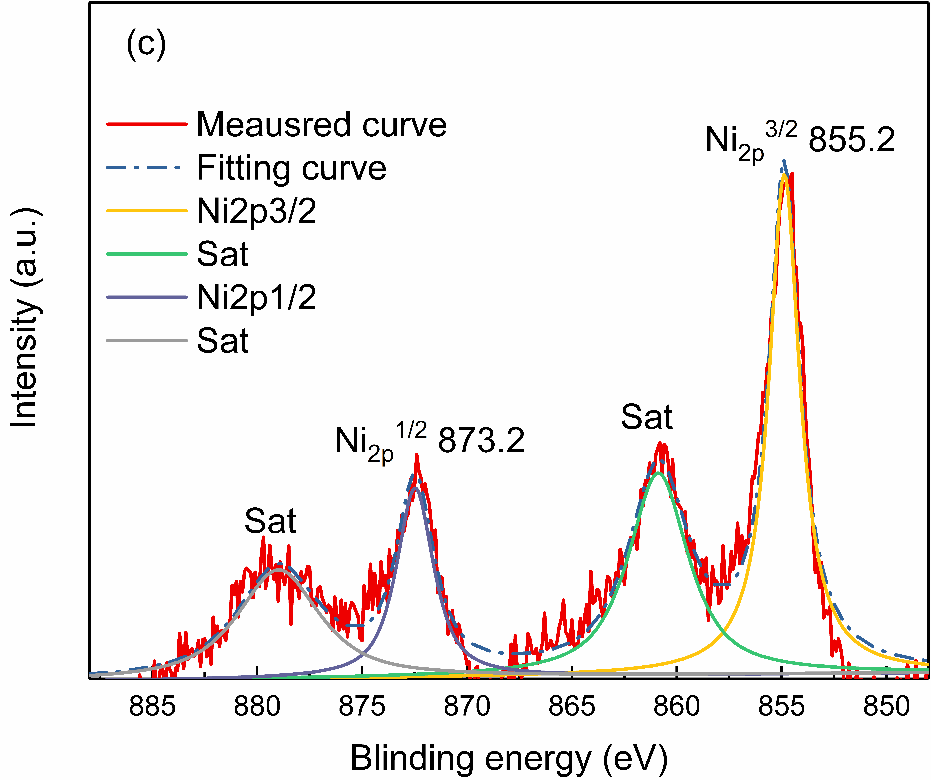
Fig.S1 (c) XPS spectrum of Ni_2p_ from the Ni/TiO_2_ catalyst





Fig.S1 (b) XPS spectrum of O_1s_ from the Ni/TiO_2_ catalyst
